# Stable hydrogen isotope variability within and among plumage tracts (*δ*^2^H_*F*_) of a migratory wood warbler

**DOI:** 10.1371/journal.pone.0193486

**Published:** 2018-04-03

**Authors:** Gary R. Graves, Seth D. Newsome, Marilyn L. Fogel

**Affiliations:** 1 Department of Vertebrate Zoology, MRC-116, National Museum of Natural History, Smithsonian Institution, Washington, District of Columbia, United States of America; 2 Center for Macroecology, Evolution and Climate, National Museum of Denmark, University of Copenhagen, Copenhagen, Denmark; 3 Biology Department, University of New Mexico, Albuquerque, New Mexico, United States of America; 4 Environmental Dynamics and Geo-Ecology Institute, University of California Riverside, Riverside, California, United States of America; Liverpool John Moores University, UNITED KINGDOM

## Abstract

Hydrogen isotope analysis of feather keratin (*δ*^2^H_*F*_) has become an essential tool for tracking movements between breeding and wintering populations of migratory birds. In particular, *δ*^2^H_*F*_ has been used to create *δ*^2^H_*F*_ isoscapes that can be used to assign the geographic origins of molt. The majority of past studies have sampled a portion of a single feather as an isotopic proxy for the entire plumage although surprisingly little is known about variation of stable isotopes within and between feather tracts of individuals in local populations. Here we examine *δ*^2^H_*F*_ variation in 24 pterylographic variables (9 primaries, 6 secondaries, 6 rectrices, and 3 patches of ventral contour feathers) in individual specimens of black-throated blue warbler (*Setophaga caerulescens*) breeding in the Big Santeetlah Creek watershed (5350 ha), southern Appalachian Mountains. By restricting our study to territorial ASY males (after second year) inhabiting a small watershed, we could focus on *δ*^2^H_*F*_ variation generated during the complete prebasic annual molt in a circumscribed population while factoring out age and sexual differences in foraging behavior, isotopic incorporation, and post-breeding dispersal. Summed within-individual variation (*δ*^2^H_*F*_) across 24 pterylographic variables ranged from 12 to 60‰ (= 21.8 ± 9.4‰), with 81% of the individuals exhibiting variation ≥ 16‰ (reproducibility of analyses was ≤ 4 ‰). Adjacent feathers in feather tracts tend to have more similar *δ*^2^H_*F*_ values than feathers grown weeks apart, consistent with the stepwise replacement of flight feathers. The pooled population sample exhibited significant *δ*^2^H_*F*_ variation in primaries (-78 to -21‰), secondaries (-80 to -17‰), rectrices (-78 to -23‰), and ventral contour feathers (-92 to -32‰). Strong year effects in *δ*^2^H_*F*_ variation were observed in each of the 24 pterylographic variables. Altitudinal effects were observed only in ventral contour feathers. The current findings demonstrate that within-individual variation (δ^2^H_*F*_) may be much greater than previously thought in migratory species that molt on or near breeding territories. Our study also highlights the need for greater pterylographic precision in research design of isotope-based studies of avian movement. Within-individual and within-population *δ*^2^H_*F*_ variation should be incorporated in geographic assignment models. In a broader context, the staggered *Staffelmauser* pattern of molt in wood warblers provides an exceptional view of the seasonal variation of hydrogen isotopes circulating in blood plasma during the six-week period of annual molt.

## Introduction

Naturally occurring stable isotopes of several light and heavy elements (i.e., H, C, O, S, and Sr) of feather keratins have been used singly or in combination to infer the foraging ecology, dispersal, and migratory connectivity of birds [1–9]. Hydrogen isotope analysis (*δ*^2^H) has figured prominently because continental isoscapes for precipitation (*δ*^2^H_*P*_) exhibit strong latitudinal gradients in North America and Europe [10–12] that are propagated through local surficial waters and food sources, which in turn are assimilated by birds and used to synthesize tissues [13, 14]. In theory, spatial variation in the isotope signatures of avian tissues should closely map the underlying isoscapes of assimilated drinking water and diet, although there are still several important unknowns such as the fluctuating variation of *δ*^2^H in diet and water available to local populations [14–16].

For species whose molt schedules are well understood, feather keratin is an ideal tissue for *δ*^2^H analysis because feathers are metabolically inert after a brief growth period (a few weeks). The isotopic composition of feathers (*δ*^2^H_*F*_) is fixed, with the exception of potentially exchangeable hydrogen that represents ~8–15% of the hydrogen budget in tissues [8, 17–19]. Several recent studies have shown that *δ*^2^H_*F*_ values for species that molt on the breeding grounds in North America and Europe typically exhibit strong correlations with amount-weighted, growing season *δ*^2^H_*P*_ [9, 20]. As a consequence, some investigators have made biological and geographical interpretations of relatively small differences in *δ*^2^H_*F*_ values (<10‰) observed among individuals and populations [21, 22]. Others have questioned whether *δ*^2^H_*F*_ values differing by <9‰ can be confidently assigned to different geographical locations [23, 24].

Pterylography is the study of feather arrangement and feather tracts of birds. Songbirds are typically plumaged with 1,000–3,000 feathers [25]. The majority of *δ*^2^H_*F*_ studies sample a single feather, typically a sub-section of a feather, as an isotopic proxy for the entire plumage of a bird [2, 22, 26–30]. Does the choice of feather have an effect on isotope-based models of movement? A few studies have made limited investigations of *δ*^2^H_*F*_ variation within individual feathers, variation among feathers within feather tracts, or variation among different feather tracts sampled from the same individual [1, 23, 26, 31–33]. Analyses that strive for an “apples to apples” comparison of individuals and populations sample the same feather (e.g., third rectrix) from every individual. This is an implicit acknowledgment that *δ*^2^H_*F*_ values may vary within and among plumage tracts and that the initiation date of molt may have an effect on *δ*^2^H_*F*_ [23, 34, 35]. In contrast, studies that pool data from different feathers within a feather tract or from feathers sampled from different feather tracts make the explicit assumption that within-individual *δ*^2^H_*F*_ variation is small compared to *δ*^2^H_*F*_ variation observed among individuals and among populations [1, 8, 30, 36].

Many studies of *δ*^2^H_*F*_ were conducted before it was recognized that temporal variability in in the isotopic composition of drinking water and diet might affect *δ*^2^H_*F*_ values of different feathers sampled from an individual. Studies of wood warblers, for example, have pooled *δ*^2^H_*F*_ data from contour (body) feathers and rectrices [1, 8], from contour feathers and primaries [4], from retrices and crown feathers [37], from the innermost and outmost rectrices [30], and from primaries and rectrices [20, 36]. In some cases, investigators have failed to specify the pterylographic origin of sampled feathers [6, 34, 38] or have vaguely referred to flight feathers [27], or have analyzed an unspecified primary [28] or rectrix [22, 26, 29, 39–41]. The extent to which pterylographic imprecision and the pooling of data from different feather tracts weakens or obfuscates research findings remains to be determined.

The *δ*^2^H_*F*_ profile of the black-throated blue warbler (*Setophaga caerulescens*) is among the best studied of North American songbirds [1, 5, 8, 42–44]. This small insectivorous warbler (body mass, 9–10 g) breeds in mixed deciduous-coniferous forests in eastern North America and winters primarily in the Greater Antilles [45–47]. Older males (ASY, after second year) show a high degree of year-to-year fidelity to breeding sites [45]. ASY males sampled from breeding populations exhibit significant latitudinal and longitudinal *δ*^2^H_*F*_ variation in ventral contour feathers [1, 8]. *δ*^2^H_*F*_ was highest in samples from the southern end of the breeding range in Georgia and lowest near the northern margins of its range in Ontario. *δ*^2^H_*F*_ also increased systematically from west (Michigan) to east (New Brunswick). The influence of altitude on *δ*^2^H_*F*_ has not been systematically studied [1, 5, 8]. In the southern Appalachian Mountains, ASY males are believed to forage within a restricted altitudinal zone centered on breeding territories not only during the early part of the breeding season [42] but also during molting period [43] as inferred from the altitudinal lapse rates (the rate at which isotope values vary with altitude) of *δ*^13^C values observed in liver, pectoral muscle, and feather keratin.

Fidelity to a relatively small breeding territory during molt should result in constrained variation of *δ*^2^H_*F*_ among feather tracts within individuals based on the premise that *δ*^2^H of food and water resources will be less variable in a narrow geographic or altitudinal zone than in a wider one. If flight feather molt begins on breeding territories and ends while individuals are engaged in post-breeding wandering to higher or lower altitudes or during migration to their wintering grounds, then *δ*^2^H_*F*_ values of ASY males could exhibit chaotic or staggered variation within feather tracts. Staggered variation in *δ*^2^H_*F*_ values within feather tracts could also result from seasonal changes in *δ*^2^H_*P*_ within territories during the course of an extended molt period. In any case, knowledge of within- and between-individual variation of *δ*^2^H_*F*_ values within circumscribed breeding populations is critical for evaluating the application of hydrogen isotopes in population “connectivity” studies [48].

In this paper, we investigate *δ*^2^H_*F*_ variation in 24 pterylographic variables (9 primaries, 6 secondaries, 6 rectrices, and 3 patches of ventral contour feathers) in the plumage of individual ASY black-throated blue warblers from a small watershed in the southern Appalachian Mountains. We characterize (*i*) *δ*^2^H_*F*_ variation within and between feather tracts in individuals; (*ii*) examine between-individual variation within the Big Santeetlah Creek breeding population; and (*iii*) document strong year effects in *δ*^2^H_*F*._ Finally, we address the implications of *δ*^2^H_*F*_ variation for connectivity studies of breeding and wintering populations of songbirds.

## Methods

### Study site

The study was conducted in western North Carolina in the Big Santeetlah Creek watershed (35° 21' N; 84° 00' W), nestled in the highest subsidiary mountain range in the Appalachian Mountains south of the Little Tennessee River [42, 43, 46, 49–52]. The watershed (680–1689 m above sea level; 5350 ha) is embedded in the largest contiguous tract of montane forest in eastern North America and supports one of the most diverse woody floras north of Mexico [53, 54]. Forestry practices since the 1920's have resulted in a mosaic of relict old-growth trees and even-aged stands of hardwood forest.

### Study species

The black-throated blue warbler is a common breeding bird between 850 m and 1450 m in forests of the southern Appalachians [46, 55, 56]. Males return to breeding territories (0.75–3.0 ha) at the lower altitudinal limit of their breeding range as early as 1 May but settlement at higher altitudes (> 1400 m) may be delayed by 2–3 weeks.

Molt patterns of the flight feathers (primaries, secondaries, rectrices) and contour plumage in the black-throated blue warbler have not been studied in detail, but like most Nearctic-Neotropical members of the genus *Setophaga* that breed in eastern North America, breeding birds are thought to undergo a complete prebasic molt (including all flight feathers) on or near their breeding territories before fall migration [57–59]. A supplementary pre-alternate molt restricted to the replacement of feathers on the head and throat occurs on the wintering grounds before spring migration [58]. In principle, *δ*^2^H_*F*_ values observed in the flight feathers of territorial birds sampled during the current breeding season (year *T)* reflect the *δ*^2^H of the food and water ingested during June, July, and August of the previous breeding season (year *T-*1). This property and the continental *δ*^2^H_*P*_ isoscape make it possible to infer the connectivity of breeding and wintering populations [1, 8].

First time breeders (SY, second year, first alternate plumage) can be distinguished in the field from older male breeders (ASY, after second year, definitive alternate plumage) by their dull primary coverts and olive margins on the alula coverts [49]. We restricted our study to territorial ASY males because older birds exhibit higher site fidelity from year to year [45]. In other words, we sampled individuals in their second or later breeding season which were likely to have bred in the Big Santeetlah Creek watershed during the previous year. Annual survivorship of males returning to breeding territories is roughly 50% [60]. By omitting females and SY males (which retain flight feathers from the juvenal plumage), we were able to focus on *δ*^2^H_*F*_ variation generated during the molting period while factoring out age and sexual differences in foraging behavior, isotopic incorporation, and post-breeding dispersal.

**Fig 1:**
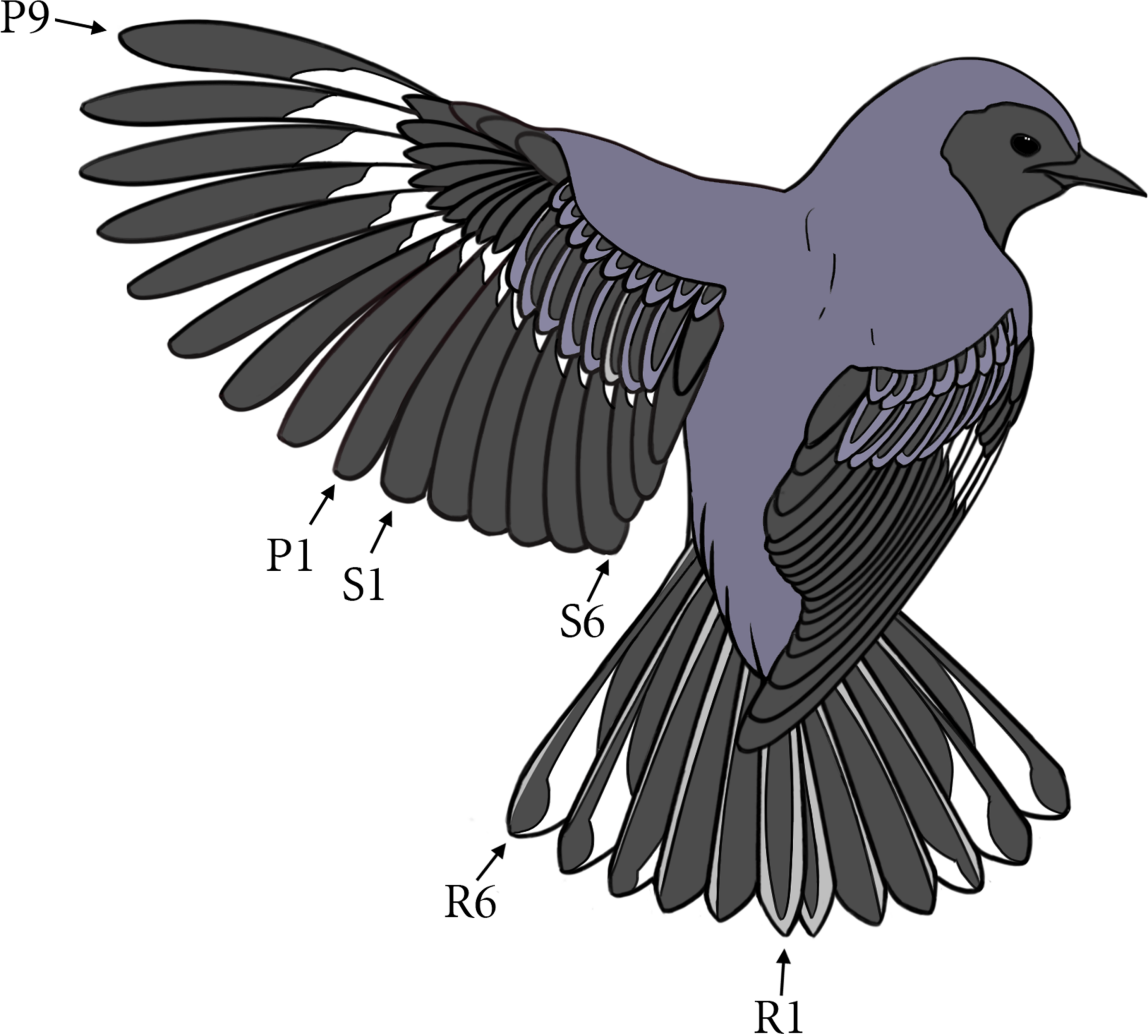
Schematic of the major flight feathers of black-throated blue warbler. Primaries (P1-P9) and secondaries (S1-S6) are numbered from the center of the wing. Rectrices (R1-R6) are numbered from the center of the tail outward. Illustration modified from an original drawing by Jacqueline Mask.

All feathers of adult black-throated blue warblers are replaced during the annual molt cycle. The principal wing feathers (Fig 1), primaries (P1–P9) and secondaries (S1–S9), are part of the alar plumage tract. Both primaries and secondaries are numbered respectively from the middle of the wing [58]. Primary molt is thought to progress in the common *Staffelmauser* pattern [61], commencing with the replacement of the first primary (P1) in the middle of the wing and followed in staggered fashion by the remaining primaries to the wingtip [57, 58]. The duration of primary molt (from loss of P1 to the full renewal of P9) is unknown in this species but it ranges from 38 to 42 days in the prairie warbler (*Setophaga discolor*) [59]. Molt of the secondaries commences with S1 in the middle of the wing and progresses toward the body [57], except for the three innermost secondaries (S4-S6) adjacent to the body, which may molt out of sequence [57, 58]. The rectrices (R1–R6), which constitute the most important component of the caudal plumage tract, are numbered outward from the center of the tail. Molt commences with the innermost rectrix (R1) and progresses rapidly outward. Molt of ventral contour feathers (upper breast, breast, lower flank) coincides with the replacement of the flight feathers.

### Specimen collection

The Smithsonian IACUC approved the research protocol. Specimen collection permits were granted by the U.S. Fish & Wildlife Service, U.S. Forest Service (Department of Agriculture), and North Carolina Wildlife Resources.

Territorial ASY males were collected 10–12 June 2013 (*n =* 15) and 16–17 June 2014 (*n =* 17) with a small bore shotgun under state and federal permits for multiple research purposes along an altitudinal transect that extended from 720 m-1540 m (above sea level). GPS coordinates were recorded at each collecting site. Altitude of collection sites was subsequently determined with Google Earth Pro. The mean altitudinal distribution of specimens in 2013 (range, 855–1491 m) and 2014 (range, 856–1394 m) was similar (ANOVA, *F*_*1*,*30*_ = 0.78; *P =* 0.38). Males collected in this study responded to playback of recorded songs and exhibited territorial behavior. Females were observed on many of the territories, but we did not systematically search for females or nests. The sampling period (10–17 June) coincided with the overlap of the fledging period for the first brood and nest building and egg laying for the second brood. Annual standardized censuses along a 14.5 km altitudinal transect indicated that the removal of males in the watershed had no demonstrable year-to-year effect on the census population.

Specimens were packaged in multiple layers of insulating tissue paper and aluminum foil and frozen within 30 minutes of death in liquid nitrogen. Voucher specimens (flat skins, partial skeletons, tissues, stomach contents) were deposited in the research collection of the National Museum of Natural History, Smithsonian Institution (USNM 647334–647338, 647340, 647342–647350, 648884–648885, 648887–648889, 648891–648901, and 648903).

### Sample preparation

We removed a section of vane (10–12 mm) from the middle of each primary (P1–P9), secondary (S1–S6), and rectrix (R1–R6) from the left or right side (but not both) of each specimen. The feather rachis was not included owing to the potential for *δ*^2^H enrichment of the rachis compared to the feather vane [23]. Given average growth rates of rectrices (3 mm/day) observed in the prairie warbler [59], our vane samples from flight feathers represent 3–4 days of growth during molt. Small contour feathers were sampled from three 5 × 5 mm patches (V1 = upper breast, V2 = breast, V3 = lower flank) on the ventral plumage tract from one side of each specimen; both vane and rachis were included in contour feather samples. All samples were washed in 2:1 chloroform:methanol to remove surface contaminants, rinsed with distilled water, and dried at 60°C. Prior to weighing, cleaned feather samples were held at the University of California Merced Stable Isotope Laboratory for at least three weeks to equilibrate possible exchangeable hydrogen. Then, approximately 0.1–0.2 mg of each sample was homogenized with surgical scissors and loaded into a 3x5 mm silver capsule for *δ*^2^H analysis. Encapsulated samples and keratin reference materials with known non-exchangeable *δ*^2^H values (see below) were kept in a drying oven at 45°C under a steady stream of N_2_ until analysis (see below).

### Stable isotope analysis

Stable isotope data are expressed as *δ* values, *δ*^2^H = 1000* [(R_sample−_ R_standard_/R_standard_)], where R_sample_ and R_standard_ are the ^2^H/^1^H of the sample and standard, respectively. The internationally accepted standard for *δ*^2^H is Vienna Standard Mean Ocean Water (V-SMOW) and the units are expressed as parts per thousand or per mil (‰). *δ*^2^H values were measured with a Thermo Scientifc thermal conversion/elemental analyzer (TC/EA) operated at 1430°C (Thermo Fisher Scientific Inc., Waltham, MA, USA) and coupled to a Thermo Scientific Delta V Plus isotope ratio mass spectrometer at the University of California, Merced (Merced, CA, USA).

Approximately 8–15% of the organically bound hydrogen in proteins can potentially exchange with hydrogen in atmospheric water vapor at ambient temperatures [18, 19]. To correct for exchangeable hydrogen in unknown samples, we used two feather keratin internal reference materials for which the *δ*^2^H composition of the non-exchangeable hydrogen fraction was determined via comparative equilibration experiments and statistical analyses identical to those described in [62]. *δ*^2^H values of the non-exchangeable fraction of hydrogen in these keratin reference materials were -55‰ (turkey feathers) and -95‰ (chicken feathers), which brackets most of the *δ*^2^H values of the warbler feathers we analyzed. Analyses of these two keratin reference materials, two oil reference materials, and pure stearic acid were used to determine the within-run precision (standard deviation) and accuracy of analyses, which was determined to be ≤ 4 ‰ relative to V-SMOW.

## Results

### Variation of *δ*^2^H_*F*_ among individuals

**Fig 2:**
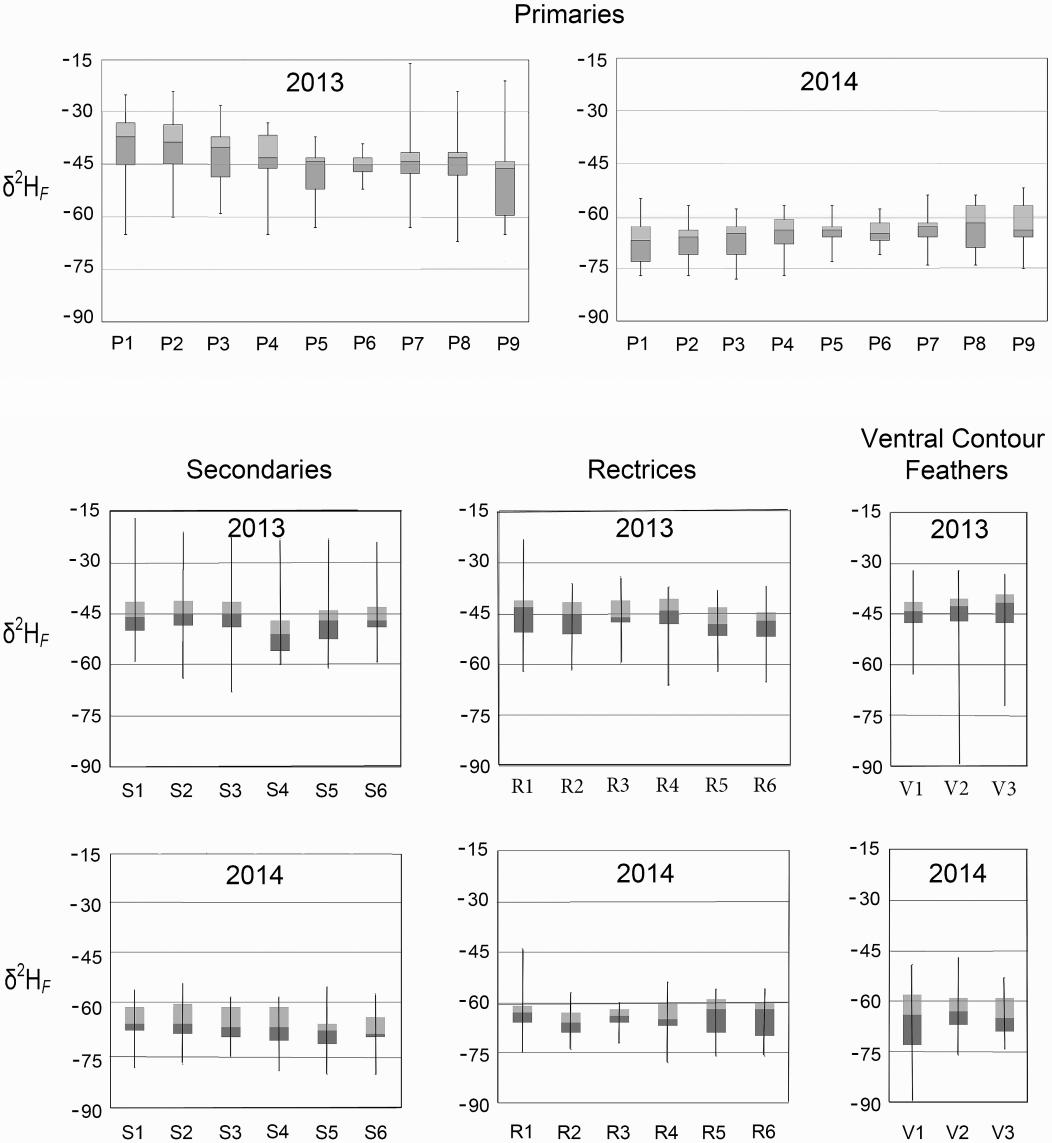
Variation of *δ*^2^H_*F*_ in plumage of black-throated blue warblers in the Big Santeetlah Creek watershed. Box plots depict minimum values, first quartile, median, third quartile, and maximum values observed among population samples for primaries (P1-P9), secondaries (S1-S6), rectrices (R1-R6), and ventral contour feathers (V1-V3). Data are presented by year (2013 and 2014).

We subjected each of the 24 pterylographic variables to a separate analysis of covariance (ANCOVA) test for year (2013 and 2014) and altitude effects on *δ*^2^H_*F*_ using the GLM (general linear model) module in SYSTAT Version 12. The alpha level (σ < 0.05) was adjusted for the number of simultaneous tests (0.05/24 = 0.002). We found differences in mean *δ*^2^H_*F*_ values between years, which are reported as Δ (equal to the mean of 2013 values minus the mean of 2014 values rounded to the nearest whole number). Mean *δ*^2^H_*F*_ values were higher in 2013 than in 2014 for all feather types (*P <* 0.0001; Fig 2): primaries (Δ = 14–28‰), secondaries (Δ = 17–21‰), rectrices (Δ = 15–19‰), and ventral contour feathers (Δ = 17–23‰). We separated the data by year in subsequent analyses. Only V1 (upper breast, ventral contour feathers) of the 24 pterylographic variables showed noticeable altitude effects in *δ*^2^H_*F*_ values (*P =* 0.013).

The cumulative range of *δ*^2^H_*F*_ values observed across all primaries (P1– P9) varied from -67 to -16‰ in 2013 and from -78 to -54‰ in 2014 (Fig 2, S1 Table). In 2013, mean *δ*^2^H_*F*_ gradually decreased from P1 (-40‰), the first primary to molt, to P9 (-49‰) at the wingtip. The opposite pattern was observed in 2014 when mean *δ*^2^H_*F*_ increased slightly from P1 (-67‰) to P9 (-63‰). In secondaries (S1–S6), *δ*^2^H_*F*_ ranged from -68 to -17‰ in 2013 and from -80 to -53‰ in 2014 (S2 Table). *δ*^2^H_*F*_ values for rectrices (R1–R6) varied from -66 to -23‰ in 2013 and from -78 to -44‰ in 2014 (S3 Table). *δ*^2^H_*F*_ values for ventral contour feathers (V1–V3) varied from -92 to -32‰ in 2013 and from -92 to -47‰ in 2014 (S4 Table).

Pearson correlation coefficients (*r*) for *δ*^2^H_*F*_ values of pairwise combinations of primaries showed significant variation in 2013 (*r* = 0.26–0.92) and 2014 (*r* = 0.19–0.86)(S5 Table). In 2013, correlations involving P1 and other primary feathers were relatively high (*r* > 0.5). In 2014, P1 correlations decreased precipitously from the middle of the wing toward the wingtip. Correlations for pairwise combinations of secondaries (S6 Table) also exhibited considerable variation in 2013 (*r* = 0.64–0.97) and in 2014 (*r =* 0.06–0.92). Pairwise correlations for rectrices were similarly varied in 2013 (*r* = 0.59–0.92) and 2014 (*r* = 0.20–0.83)(S7 Table). Pairwise correlations for ventral contour feathers exhibited a comparatively narrow range of coefficients in 2013 (*r* = 0.66–0.79) and 2014 (*r* = 0.39–0.59)(S8 Table). Overall, *δ*^2^H_*F*_ values for adjacent feathers within feather tracts exhibited higher correlation coefficients than did pairs of feathers whose growth periods overlapped less or not at all (e.g., P1 and P9)(S9 Table).

### Variation of *δ*^2^H_*F*_ within individuals

**Fig 3:**
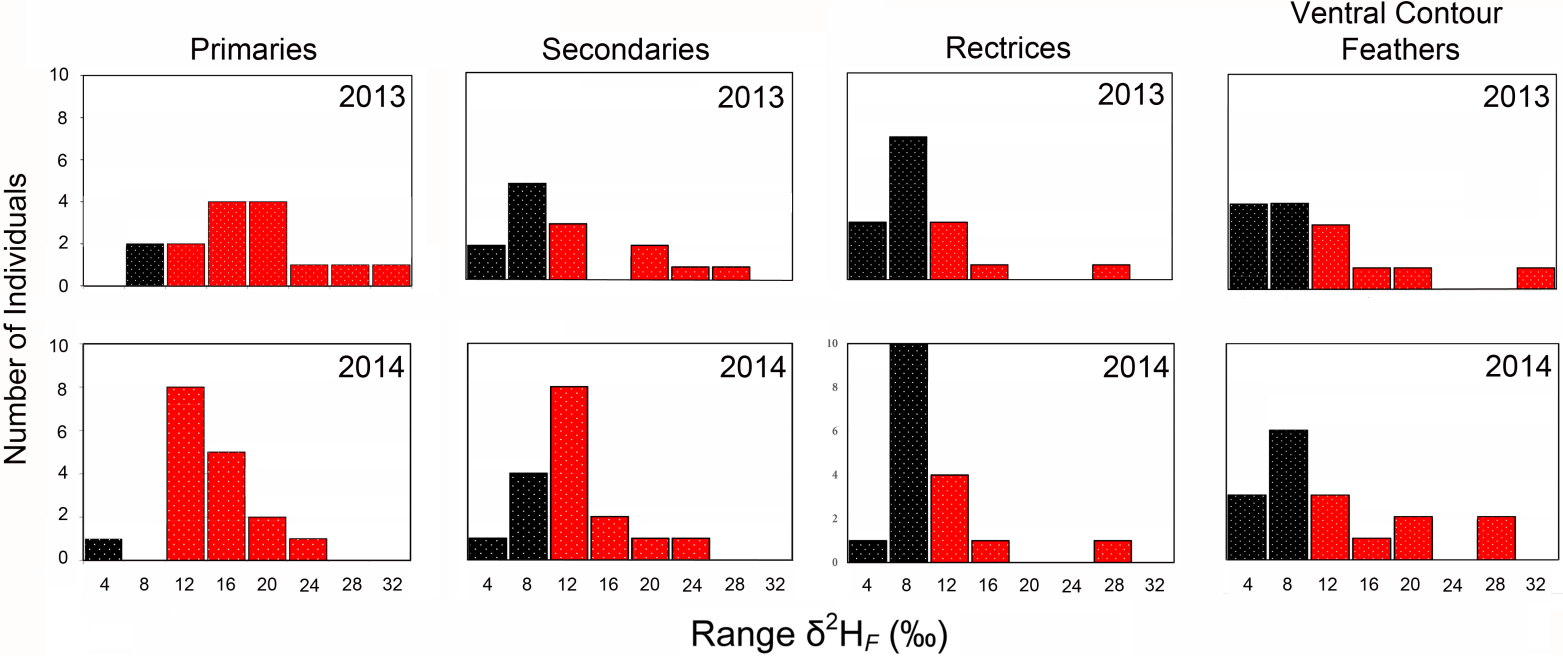
Histograms depicting the range of within-individual variation in *δ*^2^H_*F*_ in primaries (P1-P9), secondaries (S1-S6), rectrices (R1-R6), and ventral contour feathers (V1-V3) in black-throated blue warblers collected in 2013 and 2014. Individuals that exhibited a range of *δ*^2^H_*F*_ values > 8‰ (twice the maximum range of analytical variability observed within runs for laboratory reference materials) within the selected feather tracts are shown in red. Upper values for each bin are shown on the x-axis.

**Fig 4:**
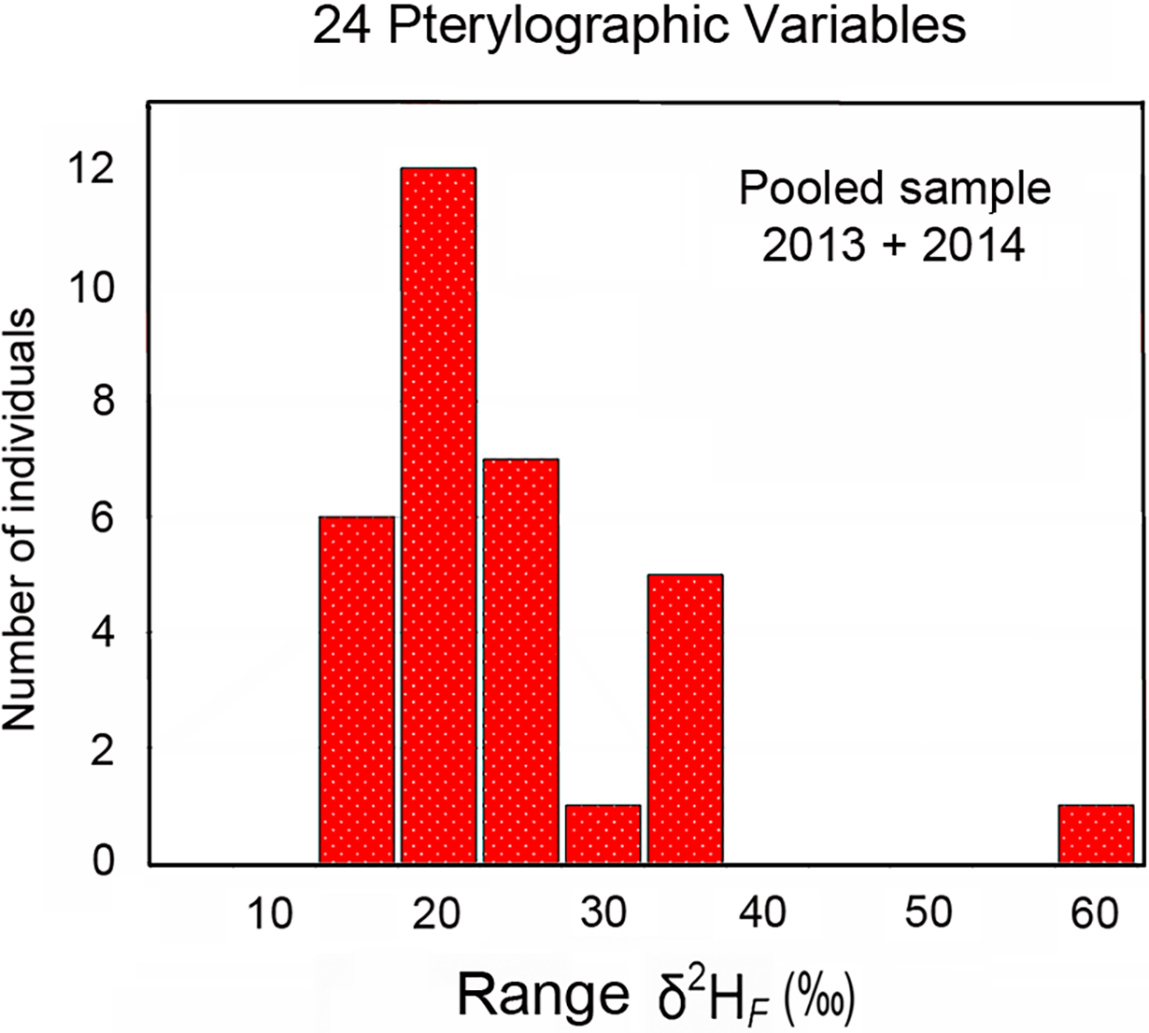
Histogram depicting the range (*δ*^2^H_*F maximum*_ - *δ*^2^H_*F minimum*_) of within-individual *δ*^2^H_*F*_ observed across all 24 pterylographic variables in black-throated blue warblers. Individuals that exhibited a range of *δ*^2^H_*F*_ variation > 8 ‰ are shown in red. Upper values for each bin are shown on the x-axis.

Within-individual variation of *δ*^2^H_*F*_ exceeded analytical precision (± 4‰) in one or more of the three feather tracts (alar, caudal, ventral) in all specimens sampled in 2013 and 2014 (Fig 3). The range of *δ*^2^H_*F*_ values (*δ*^2^H_*F maximum*_ - *δ*^2^H_*F minimum*_) observed in primaries sampled from individual birds was substantial in 2013 (8–31‰) and in 2014 (4–21‰). The range of *δ*^2^H_*F*_ observed in secondaries was also substantial in 2013 (2–26‰) and in 2014 (4–22‰). Fewer individuals exhibited *δ*^2^H_*F*_ variation in rectrices that exceeded analytical precision, but *δ*^2^H_*F*_ still varied extensively in 2013 (4–25‰) and in 2014 (3–26‰). The range of *δ*^2^H_*F*_ observed in ventral contour feathers was also considerable in 2013 (1–30‰) and in 2014 (2–27‰). Summed across all 24 pterylographic variables, the range of within-individual variation in *δ*^2^H_*F*_ was large in 2013 (14–60‰; = 23.7 ± 11.7‰) and in 2014 (12–33‰; = 20.2 ± 6.7‰)(Fig 4). The range of *δ*^2^H_*F*_ values observed among the pooled flight feathers (primaries, secondaries, and rectrices) within individuals was uncorrelated (*P >* 0.05) with altitude of the collecting site.

## Discussion

Our study provides the first fine-grained view of within-individual *δ*^2^H_*F*_ variation in the plumage of wild songbirds. Perhaps the most significant finding was the substantial *δ*^2^H_*F*_ heterogeneity that occurs within and between feather tracts of individual black-throated blue warblers. For example, the range of *δ*^2^H_*F*_ values observed across primaries (P1–P9) varied from 4 to 31‰ (= 15 ± 6‰). The range of within-individual variation observed across all 24 pterylographic variables (Fig 4) varied from 12 to 60‰ (= 22 ± 9‰), with 81% of the individuals exhibiting ranges ≥ 16‰. Given the paucity of published data on within-individual heterogeneity (*δ*^2^H_*F*_), it is impossible to say whether the *δ*^2^H_*F*_ variation observed in black-throated blue warblers is unusual for a songbird species that molts on the breeding grounds. Comparable *δ*^2^H_*F*_ data from a small collection of Swainson’s thrush (*Catharus ustulatus*; *n =* 9) collected in Quebec [23] exhibited within-feather heterogeneity of 2 to 17‰ (= 5 ± 5‰) based on three subsamples from a single flight feather per specimen. If the sampling had been expanded to include feathers from other feather tracks, the cumulative within-individual variation would likely have been larger.

The general pattern that emerged in the correlation matrices of pairwise combinations of feathers in the current study was that *δ*^2^H_*F*_ values of adjacent feathers within a feather tract tended to be more similar than those of randomly chosen pairs of feathers. This observation is consistent with the stepwise replacement of flight feathers in wood warblers [58, 59]. Feathers grown at or near the same time tend to have more similar *δ*^2^H_*F*_ values than feathers grown weeks apart. This pattern and the more general observation of within-individual heterogeneity (*δ*^2^H_*F*_) could be caused by several covarying factors: (*i*) seasonal variation in *δ*^2^H_*P*_ [11] propagated through drinking water and the insect diet of warblers during the 6-week molting period; (*ii*) physiological variation in isotopic discrimination linked to variation in dietary macromolecular content, the contribution of metabolic water to tissue *δ*^2^H, and the potential for isotopic routing during molt and pre-migratory conditioning (*iii*) seasonal variation in heat stress and evaporative cooling in breeding and molting individuals resulting in isotopic enrichment of body water [33, 63]; (*iv*) local post-breeding wandering [64] in isotopically heterogeneous habitats when molt is still active; or (*v*) replacement of feathers lost accidentally during migration or on the wintering grounds rather than during the summer breeding season [58, 65].

Lacking data on the location, movement, diet, and drinking water of individuals during the molting period, we are unable to identify the exact causes of within-individual heterogeneity of *δ*^2^H_*F*_ in our breeding population sample. Suffice to say, within-individual heterogeneity of *δ*^2^H_*F*_ was uncorrelated with the altitude of the individual’s territory at the time of collection. Additionally, we observed only a single individual in which *δ*^2^H_*F*_ values of adjacent primaries or rectrices (the feather tracts most frequently analyzed in previous studies) differed to an extent (> 20‰) that suggested the loss and regrowth of a feather during migration or on the Antillean wintering grounds (see Fig 2 in reference 34).

A second prominent finding was that the range of population-level heterogeneity of *δ*^2^H_*F*_ (76‰) observed in the Santeetlah Creek watershed across the 24 pterylographic variables (*n =* 32 specimens) was similar in extent to *δ*^2^H_*F*_ variation in ventral contour feathers of black-throated blue warblers collected at multiple breeding locations in the Appalachians from 35° to 44° N [1, 8]. Basiolli (2008) observed a slightly smaller range of within-population heterogeneity (63‰) in rectrices of black-throated blue warblers that returned to breeding territories in New Hampshire. Basiolli’s data (*n* = 43 individuals) included three outliers that were thought to have molted south of the breeding grounds [41]. Collectively, these data indicate that within-population *δ*^2^H_*F*_ variation contributes a substantial fraction of the total variance in *δ*^2^H_*F*_ observed in black-throated blue warblers across its geographic range. Causes of population-level variation would include the roster of factors previously mentioned for within-individual heterogeneity as well as tangible differences in the water and diet resources present in non-overlapping breeding territories in both local populations and in geographically separated populations.

A third significant but perhaps less surprising finding was a strong year effect in *δ*^2^H_*F*_ in each of the 24 pterylographic variables. *δ*^2^H_*F*_ values averaged 20 ‰ higher in 2013 than in 2014 (S1 Table), suggesting a systemic environmental cause for the difference. Strong year effects in carbon isotopes (*δ*^13^C) of feather keratin was reported by [43], as well as pectoral muscle and liver *δ*^13^C values [42] collected from black-throated blue warblers in the same watershed [43]. Whatever the cause, significant year-to-year fluctuations in *δ*^2^H_*F*_ and *δ*^13^C_*F*_ within a local watershed indicate that temporal effects clearly influence analyses of continent-wide isotope gradients in feather keratin if geographically dispersed populations are sampled in different years. Previous geographic studies of *δ*^2^H_*F*_ variation in black-throated blue warblers did, in fact, pool population samples collected during several breeding seasons [1, 8]. The collection of specimens at a number of widely separated locations within the warbler’s extensive breeding range during a single (brief) breeding season would be logistically difficult. Both of these studies were completed years before it was widely recognized that annual variation in *δ*^2^H_*P*_ could override the long-term latitudinal variation in *δ*^2^H_*P*_ signal that is used to discriminate among local bird populations via *δ*^2^H_*F*_ analysis of growing feathers [35, 66–68]. Our study adds additional support to recommendations that year effects should be factored into geographic assignment models [68].

## Conclusions

We draw several conclusions from the exceptional *δ*^2^H_*F*_ variability observed in the Santeetlah Creek population of black-throated blue warblers. First, accuracy of geographic assignment tests in studies of migratory connectivity hinges on the accuracy of transfer functions to convert *δ*^2^H*p* into *δ*^2^H_*F*_ [9]. Transfer functions, which implicitly incorporate a variety of abiotic, physiological, and ecological processes are ideally estimated by documenting the hydrogen isotope discrimination factor between feather keratins and local precipitation at each of many known molting areas for each study species. In practice, species-specific transfer functions were traditionally based on the intercept of linear regression of *δ*^2^H_*F*_ on *δ*^2^H*p* of the pooled sample collected at many locations [6, 9]. More recent models have incorporated data on foraging guild, foraging substrate, nesting substrate, diet, and age class among other variables [20, 35]. Conspicuously absent from the latest iterations of both transfer functions and geographic assignment models is the explicit consideration of population-specific data on within-individual heterogeneity of *δ*^2^H_*F*_, the observed variation within and between feather tracts of individual birds.

Second, the finding of high levels of within-individual variation in *δ*^2^H_*F*_ in black-throated blue warblers emphasizes the necessity of homologous sampling when studies aim to compare *δ*^2^H_*F*_ variation among populations but are limited to a single feather sample per individual. Prebasic molt is a prolonged process that requires up to six weeks to complete in wood warblers. Based on our data, the first and last feathers grown during a single molt cycle on the breeding grounds would in most individuals be classified as originating from molting sites that are separated by several degrees of latitude using the stringency benchmarks commonly employed in *δ*^2^H_*F*_ studies of avian movement [21, 22, 26, 38, 69]. In any event, we recommend the incorporation of within-individual variation of *δ*^2^H_*F*_ in geographic assignment models. In a broader context, the *Staffelmauser* pattern of molt in wood warblers provides a unique window on the seasonal variation of hydrogen isotopes circulating in blood plasma during the six week period of annual molt on the breeding grounds.

## Supporting information

S1 Table Summary statistics for *δ*^2^H_*F*_ values for primaries (P1-P9). (PDF)

S2 Table Summary statistics for *δ*^2^H_*F*_ values for secondaries (S1-S6). (PDF)

S3 Table Summary statistics for *δ*^2^H_*F*_ values for rectrices (R1-R6). (PDF)

S4 Table Summary statistics for *δ*^2^H_*F*_ values for ventral contour feathers (V1-V3). (PDF)

S5 Table Pearson correlation coefficients for *δ*^2^H_*F*_ values for pairwise combinations of primaries (P1-P9). (PDF)

S6 Table Pearson correlation coefficients for *δ*^2^H_*F*_ values for pairwise combinations of secondaries (S1-S6). (PDF)

S7 Table Pearson correlation coefficients for *δ*^2^H_*F*_ values for pairwise combinations of rectrices (R1-R6). (PDF)

S8 Table Pearson correlation coefficients for *δ*^2^H_*F*_ values for pairwise combinations of ventral contour feathers (V1-V3). (PDF)

S9 Table Raw *δ*^2^H_*F*_ values. (PDF)
