# Supplementary material for: Stable hydrogen isotope variability within and among plumage tracts (δ2HF) of a migratory wood warbler
Source: PLoS One. 2018 Apr 3;13(4):e0193486. doi: 10.1371/journal.pone.0193486 (PMC5882105; doi:10.1371/journal.pone.0193486)
Supplement: S1 Table — (PDF) [file pone.0193486.s001.pdf]

Stable Hydrogen Isotope Variability within and among Plumage Tracts (δ^2H_F) of a Migratory Wood Warbler

S1 Table. Summary statistics for δ^2H_F values for primaries (P1-P9) for black-throated blue warblers collected in the Big Santeetlah Creek watershed in 2013 and 2014.

2013

	P1	P2	P3	P4	P5	P6	P7	P8	P9
n	15	14	15	15	15	13	15	15	15
Minimum	-65	-60	-59	-65	-63	-52	-63	-67	-65
Maximum	-25	-24	-28	-33	-37	-39	-16	-24	-21
Range (max-min)	40	36	31	32	26	13	47	43	44
Mean	-39.7	-39.0	-42.2	-43.4	-47.0	-44.8	-44.9	-44.9	-48.9
Standard deviation	11.1	9.8	8.9	8.3	7.9	3.8	11.0	9.4	11.6

2014

	P1	P2	P3	P4	P5	P6	P7	P8	P9
N	17	17	17	17	17	17	17	17	17
Minimum	-77	-77	-78	-77	-73	-71	-74	-74	-75
Maximum	-55	-57	-58	-57	-57	-58	-54	-54	-52
Range (max-min)	22	20	20	20	16	13	20	20	23
Mean	-66.6	-66.6	-67.2	-64.9	-64.4	-64.2	-64.0	-62.9	-63.0
Standard deviation	7.0	5.7	5.6	5.1	3.8	3.6	5.6	6.6	6.6
