# Supplementary material for: Stable hydrogen isotope variability within and among plumage tracts (δ2HF) of a migratory wood warbler
Source: PLoS One. 2018 Apr 3;13(4):e0193486. doi: 10.1371/journal.pone.0193486 (PMC5882105; doi:10.1371/journal.pone.0193486)
Supplement: S2 Table — (PDF) [file pone.0193486.s002.pdf]

Stable Hydrogen Isotope Variability within and among Plumage Tracts (δ^2H_F) of a Migratory Wood Warbler

S2 Table. Summary statistics for δ^2H_F values for secondaries (S1-S6) for black-throated blue warblers collected in the Big Santeetlah Creek watershed in 2013 and 2014.

2013

	S1	S2	S3	S4	S5	S6
N	15	14	15	13	15	13
Minimum	-59	-64	-68	-60	-61	-59
Maximum	-17	-21	-22	-23	-23	-24
Range (max-min)	42	43	46	37	38	35
Mean	-43.2	-45.2	-45.9	-49.2	-47.7	-46.2
Standard deviation	11.0	9.5	9.8	9.5	9.4	8.3

2014

	S1	S2	S3	S4	S5	S6
N	17	17	17	17	17	17
Minimum	-78	-77	-75	-79	-80	-80
Maximum	-55	-53	-57	-57	-54	-56
Range (min-max)	23	24	18	22	26	24
Mean	-64.5	-63.8	-65.0	-66.1	-67.1	-67.3
Standard deviation	5.9	7.2	6.1	6.3	5.9	5.9
