# Supplementary material for: Stable hydrogen isotope variability within and among plumage tracts (δ2HF) of a migratory wood warbler
Source: PLoS One. 2018 Apr 3;13(4):e0193486. doi: 10.1371/journal.pone.0193486 (PMC5882105; doi:10.1371/journal.pone.0193486)
Supplement: S3 Table — (PDF) [file pone.0193486.s003.pdf]

Stable Hydrogen Isotope Variability within and among Plumage Tracts (δ^2H_F) of a Migratory Wood Warbler

S3 Table. Summary statistics for δ^2H_F values for rectrices (R1-R6) for black-throated blue warblers collected in the Big Santeetlah Creek watershed in 2013 and 2014.

2013

	R1	R2	R3	R4	R5	R6
N	15	15	15	15	15	14
Minimum	-62	-61	-60	-66	-62	-65
Maximum	-23	-36	-34	-37	-38	-37
Range (min-max)	39	25	26	29	24	28
Mean	-45.1	-46.6	-45.9	-46.6	-48.6	-48.2
Standard deviation	9.5	7,2	6,6	8.3	7.0	7.1

2014

	R1	R2	R3	R4	R5	R6
N	17	17	17	17	17	17
Minimum	-75	-74	-72	-78	-76	-76
Maximum	-44	-57	-60	-54	-56	-56
Range (min-max)	31	17	12	24	20	20
Mean	-62.9	-65.7	-64.4	-64.9	-63.9	-64.4
Standard deviation	6.8	4.9	3.7	6.1	5.9	6.2
