# Supplementary material for: Stable hydrogen isotope variability within and among plumage tracts (δ2HF) of a migratory wood warbler
Source: PLoS One. 2018 Apr 3;13(4):e0193486. doi: 10.1371/journal.pone.0193486 (PMC5882105; doi:10.1371/journal.pone.0193486)
Supplement: S4 Table — (PDF) [file pone.0193486.s004.pdf]

Stable Hydrogen Isotope Variability within and among Plumage Tracts (δ^2H_F) of a Migratory Wood Warbler

S4 Table. Summary statistics for δ^2H_F values for ventral contour feathers (V1-V3) for black-throated blue warblers collected in the Big Santeetlah Creek watershed in 2013 and 2014.

2013

	V1	V2	V3
N	14	14	14
Minimum	-62	-92	-72
Maximum	-32	-32	-33
Range (min-max)	30	60	39
Mean	-44.2	-45.9	-45.4
Standard deviation	7.4	14.2	10.4

2014

	V1	V2	V3
N	17	17	17
Minimum	-92	-76	-74
Maximum	-49	-47	-53
Range (min-max)	43	29	21
Mean	-66.7	-62.4	-64.7
Standard deviation	11.5	7.1	6.1
