# Supplementary material for: Stable hydrogen isotope variability within and among plumage tracts (δ2HF) of a migratory wood warbler
Source: PLoS One. 2018 Apr 3;13(4):e0193486. doi: 10.1371/journal.pone.0193486 (PMC5882105; doi:10.1371/journal.pone.0193486)
Supplement: S7 Table — (PDF) [file pone.0193486.s007.pdf]

Stable Hydrogen Isotope Variability within and among Plumage Tracts (δ^2H_F) of a Migratory Wood Warbler

S7 Table. Pearson correlation coefficients (*r*) for δ^2H_F values of pairwise combinations of rectrices (R1-R6) sampled within individual black-throated blue warblers. 2013 males (n = 14-15) above the diagonal and 2014 males (*n* = 17) below the diagonal. Correlation coefficients $\geq$ 0.80 are shaded with yellow.

	R1	R2	R3	R4	R5	R6
R1		0.74	0.59	0.77	0.61	0.59
R2	0.40		0.92	0.90	0.87	0.85
R3	0.21	0.82		0.92	0.78	0.85
R4	0.42	0.73	0.68		0.76	0.86
R5	0.31	0.83	0.83	0.76		0.89
R6	0.20	0.61	0.65	0.48	0.83	
