# Supplementary material for: Stable hydrogen isotope variability within and among plumage tracts (δ2HF) of a migratory wood warbler
Source: PLoS One. 2018 Apr 3;13(4):e0193486. doi: 10.1371/journal.pone.0193486 (PMC5882105; doi:10.1371/journal.pone.0193486)
Supplement: S8 Table — (PDF) [file pone.0193486.s008.pdf]

Stable Hydrogen Isotope Variability within and among Plumage Tracts (δ^2H_F) of a Migratory Wood Warbler

S8 Table. Pearson correlation coefficients (*r*) for δ^2H_F values of pairwise combinations of ventral contour feathers (V1-V3) sampled within individual black-throated blue warblers. 2013 males (n = 14) above the diagonal and 2014 males (*n* = 17) below the diagonal.

	V1	V2	V3
V1		0.76	0.66
V2	0.41		0.79
V3	0.39	0.55	
