# Supplementary material for: Stable hydrogen isotope variability within and among plumage tracts (δ2HF) of a migratory wood warbler
Source: PLoS One. 2018 Apr 3;13(4):e0193486. doi: 10.1371/journal.pone.0193486 (PMC5882105; doi:10.1371/journal.pone.0193486)
Supplement: S9 Table — (PDF) [file pone.0193486.s009.pdf]

S9 Table. δ^2H_F values for black-throated blue warblers collected in 2013 and 2014 in the Big Santeetlah Creek watershed. Primaries (P1-P9). Secondaries (S1-S6). Rectrices (R1-R6). Ventral contour feathers (V1-V3).

**USNM**	**Year**	**Altitude**	**P1**	**P2**	**P3**	**P4**	**P5**	**P6**	**P7**	**P8**	**P9**	**S1**	**S2**	**S3**	**S4**	**S5**	**S6**
647334	2013	1263	-65	-60	-59	-65	-63	NA	-63	-67	-65	-59	-64	-68	-56	-61	-49
647335	2013	1124	-56	-51	-54	-47	-58	-52	-60	-47	-58	-53	-54	-49	NA	-58	-59
647336	2013	1123	-47	-44	-48	-54	-58	NA	-58	-57	-64	-51	NA	-56	-57	-61	NA
647337	2013	991	-26	-28	-29	-37	-39	-39	-38	-39	-39	-41	-41	-40	-42	-44	NA
647338	2013	992	-37	-41	-37	-35	-43	-47	-47	-42	-43	-43	-44	-45	NA	-43	-43
647340	2013	855	-45	-39	-43	-42	-45	-39	-43	-42	-49	-48	-47	-46	-47	-40	-42
647342	2013	875	-33	-35	-49	-43	-52	-44	-42	-43	-44	-42	-41	-41	-52	-47	-45
647343	2013	1202	-28	-26	-28	-33	-43	-42	-41	-41	-45	-46	-41	-41	-60	-44	-45
647344	2013	1390	-35	NA	-35	-35	-52	-50	-46	-43	-44	-50	-47	-44	-51	-50	-51
647345	2013	1381	-41	-37	-38	-44	-43	-45	-43	-43	-45	-25	-43	-44	-48	-48	-49
647346	2013	1381	-33	-33	-38	-44	-43	-43	-40	-39	-46	-46	-42	-42	-48	-47	-47
647347	2013	1045	-35	-45	-48	-48	-44	-47	-48	-47	-47	-45	-49	-49	-46	-45	-43
647348	2013	1247	-25	-24	-40	-36	-47	-43	-16	-24	-21	-17	-21	-22	-23	-23	-24
647349	2013	1491	-44	-45	-50	-43	-37	-46	-44	-49	-62	-32	-46	-49	-58	-52	-48
647350	2013	1172	-45	-38	-37	-45	-38	-45	-45	-51	-61	-50	-53	-53	-52	-53	-56
648884	2014	875	-55	-57	-58	-59	-62	-58	-54	-54	-53	-59	-60	-59	-57	-65	-63
648885	2014	856	-74	-73	-70	-70	-64	-66	-62	-63	-63	-65	-65	-66	-74	-74	-76
648887	2014	1111	-73	-71	-68	-68	-67	-63	-62	-57	-60	-68	-62	-57	-58	-62	-62
648888	2014	1309	-73	-77	-78	-72	-73	-67	-74	-74	-64	-72	-68	-70	-70	-72	-72
648889	2014	1295	-64	-66	-64	-64	-63	-66	-63	-62	-65	-67	-59	-69	-71	-71	-74
648891	2014	1394	-66	-71	-76	-68	-64	-66	-72	-66	-63	-66	-68	-72	-68	-73	-68
648892	2014	1394	-69	-66	-64	-62	-63	-58	-56	-57	-52	-60	-54	-58	-60	-54	-56
648893	2014	1384	-73	-72	-74	-65	-59	-62	-62	-67	-65	-59	-65	-60	-65	-67	-68
648894	2014	1381	-55	-58	-65	-57	-57	-61	-60	-56	-66	-60	-65	-66	-71	-68	-67
648895	2014	1372	-55	-59	-65	-60	-61	-62	-64	-56	-56	-60	-53	-59	-69	-66	-69
648896	2014	1324	-61	-66	-63	-68	-69	-67	-65	-69	-75	-55	-77	-69	-70	-67	-68
648897	2014	1338	-67	-66	-71	-77	-66	-71	-74	-73	-73	-72	-77	-75	-79	-80	-80
648898	2014	1330	-63	-62	-63	-61	-64	-65	-68	-70	-64	-60	-71	-68	-64	-63	-64
648899	2014	1072	-66	-67	-71	-62	-63	-61	-63	-59	-57	-63	-55	-60	-58	-65	-62
648900	2014	1123	-77	-73	-69	-65	-65	-67	-60	-56	-70	-67	-57	-62	-64	-67	-68
648901	2014	1123	-68	-64	-60	-61	-66	-68	-63	-60	-57	-78	-62	-60	-59	-60	-63
648903	2014	1171	-73	-65	-63	-64	-69	-64	-66	-70	-68	-66	-67	-75	-66	-67	-64

S9 Table extended

R1	R2	R3	R4	R5	R6	V1	V2	V3
-62	-61	-60	-66	-61	-65	-45	-51	-45
-52	-54	-52	-53	-56	-52	-44	-49	-59
-59	-58	-56	-62	-53	-55	-50	-39	-56
-38	-39	-40	-44	-41	NA	NA	NA	NA
-40	-41	-41	-40	-47	-51	-41	-43	-38
-49	-43	-39	-38	-38	-37	-32	-32	-33
-48	-47	-46	-48	-50	-47	-35	-42	-42
-43	-44	-46	-46	-48	-44	-44	-44	-41
-43	-48	-46	-44	-49	-46	-48	-44	-39
-43	-42	-47	-44	-44	-46	-50	-41	-38
-38	-36	-34	-37	-42	-39	-42	-41	-39
-42	-41	-41	-40	-42	-43	-36	-36	-46
-23	-46	-48	-41	-47	-47	-46	-40	-40
-44	-45	-46	-48	-49	-48	-62	-92	-72
-53	-54	-47	-48	-62	-55	-44	-48	-48
-63	-59	-62	-60	-57	-58	-55	-52	-53
-67	-65	-66	-65	-62	-64	-73	-76	-69
-61	-67	-68	-65	-63	-62	-58	-58	-64
-70	-74	-72	-78	-76	-72	-90	-70	-64
-65	-67	-61	-59	-62	-60	-92	-65	-69
-44	-70	-69	-67	-70	-70	-66	-65	-72
-57	-60	-62	-60	-56	-56	-63	-54	-57
-57	-57	-61	-54	-58	-62	-61	-59	-59
-62	-66	-64	-59	-62	-61	-73	-62	-65
-60	-61	-60	-62	-59	-62	-65	-61	-64
-75	-70	-66	-73	-69	-66	-57	-63	-68
-71	-74	-71	-69	-72	-76	-73	-67	-70
-61	-63	-62	-66	-64	-73	-63	-60	-57
-62	-63	-61	-62	-59	-58	-49	-67	-58
-66	-69	-63	-67	-60	-60	-58	-67	-74
-63	-65	-64	-74	-67	-61	-64	-47	-65
-66	-67	-65	-63	-71	-74	-74	-68	-72
